# Prediction of Mortality in Patients with Isolated Traumatic Subarachnoid Hemorrhage Using a Decision Tree Classifier: A Retrospective Analysis Based on a Trauma Registry System

**DOI:** 10.3390/ijerph14111420

**Published:** 2017-11-22

**Authors:** Cheng-Shyuan Rau, Shao-Chun Wu, Peng-Chen Chien, Pao-Jen Kuo, Yi-Chun Chen, Hsiao-Yun Hsieh, Ching-Hua Hsieh

**Affiliations:** 1 Department of Neurosurgery, Kaohsiung Chang Gung Memorial Hospital, Kaohsiung 83301, Taiwan; ersh2127@cloud.cgmh.org.tw; 2 Department of Anesthesiology, Kaohsiung Chang Gung Memorial Hospital, Kaohsiung 83301, Taiwan; shaochunwu@gmail.com; 3 Department of Plastic Surgery, Kaohsiung Chang Gung Memorial Hospital, Kaohsiung 83301, Taiwan; venu_chien@hotmail.com (P.-C.C.); bow110470@gmail.com (P.-J.K.); libe320@yahoo.com.tw (Y.-C.C.); sylvia19870714@hotmail.com (H.-Y.H.)

**Keywords:** traumatic brain injury, subarachnoid hemorrhage, traumatic subarachnoid hemorrhage, mortality, decision tree

## Abstract

*Background:* In contrast to patients with traumatic subarachnoid hemorrhage (tSAH) in the presence of other types of intracranial hemorrhage, the prognosis of patients with isolated tSAH is good. The incidence of mortality in these patients ranges from 0–2.5%. However, few data or predictive models are available for the identification of patients with a high mortality risk. In this study, we aimed to construct a model for mortality prediction using a decision tree (DT) algorithm, along with data obtained from a population-based trauma registry, in a Level 1 trauma center. *Methods:* Five hundred and forty-five patients with isolated tSAH, including 533 patients who survived and 12 who died, between January 2009 and December 2016, were allocated to training (*n* = 377) or test (*n* = 168) sets. Using the data on demographics and injury characteristics, as well as laboratory data of the patients, classification and regression tree (CART) analysis was performed based on the Gini impurity index, using the rpart function in the rpart package in R. *Results:* In this established DT model, three nodes (head Abbreviated Injury Scale (AIS) score ≤4, creatinine (Cr) <1.4 mg/dL, and age <76 years) were identified as important determinative variables in the prediction of mortality. Of the patients with isolated tSAH, 60% of those with a head AIS >4 died, as did the 57% of those with an AIS score ≤4, but Cr ≥1.4 and age ≥76 years. All patients who did not meet the above-mentioned criteria survived. With all the variables in the model, the DT achieved an accuracy of 97.9% (sensitivity of 90.9% and specificity of 98.1%) and 97.7% (sensitivity of 100% and specificity of 97.7%), for the training set and test set, respectively. *Conclusions:* The study established a DT model with three nodes (head AIS score ≤4, Cr <1.4, and age <76 years) to predict fatal outcomes in patients with isolated tSAH. The proposed decision-making algorithm may help identify patients with a high risk of mortality.

## 1. Introduction

Traumatic subarachnoid hemorrhage (tSAH) is frequently observed in patients with head injuries [1]. In patients visiting the emergency department (ED) for the treatment of blunt head injury, tSAH was found to be the second most frequent consequence of traumatic brain injury (TBI) [2] and occurred in 33–60% of patients after moderate or severe TBI [1,3,4,5]. It has been shown that tSAH is a marker of a more severe initial injury, indicating greater mechanical forces and intracranial deformation [6]. In the presence of tSAH, symptomatic cerebral vasospasm may develop in around 20% of patients with severe TBI [7,8].

Isolated tSAH is defined as the exclusive presence of tSAH in the absence of any other traumatic radiographic intracranial pathology. In contrast to those patients with tSAH in the presence of other intracranial hemorrhage, the prognosis of patients with isolated tSAH is good [9,10]. In patients with mild TBI (Glasgow Coma Scale (GCS) score ≥13) and isolated tSAH, no neurologic decline or need for neurosurgical procedures was observed [11,12,13,14,15]. In a retrospective study evaluating isolated tSAH in patients with mild TBI (GCS 13–15), of 67 patients, only 1 patient (1.5%) experienced neurological deterioration, and not a single patient required neurosurgical intervention [16]. In a meta-analysis study, the cumulative incidences of radiographic progression and eventual neurosurgical intervention after isolated tSAH were 5.76% (95% confidence interval (CI) 1.18–12.9%) and 0.0017% (95% CI 0–0.39%), respectively [10]. In the same meta-analysis study, among eight studies with a total of 873 patients with isolated tSAH, the incidence of mortality ranged from 0–2.5%, with a cumulative incidence of 0.60% across all the included studies (95% CI 0.09–1.4%) [10].

A previously conducted study recommended a protocol which did not require transfer for neurosurgical consultation for patients with isolated tSAH and a GCS score of 15 [10,17]. However, for most of the patients found to have an isolated tSAH on the CT scan, few data are available to help in the identification of high-risk individuals and guide physicians on which patients will likely need further evaluation and treatment. Despite the frequency of neurosurgical consultation, only a minority of patients may actually undergo neurosurgical intervention. Two common prediction models (the International Mission for Prognosis and Analysis of Clinical Trials in Traumatic Brain Injury (IMPACT)) and Corticosteroid Randomization after Significant Head Injury (CRASH)), based on large clinical trial datasets, have shown good discrimination and accurate outcome predictions for patients with TBI [18,19,20]. However, these two models lack the precision required for use at the individual patient level [21,22], and are not suitable to be applied in the management of patients with isolated tSAH.

The decision tree (DT) is a machine learning model, and is composed of decision rules based on optimal feature cutoff values that recursively split independent variables into different groups, and predict an outcome in a hierarchical manner [17,23,24]. To define the variables that could identify individuals at a risk for mortality from among patients with isolated tSAH, we aimed to construct a model for mortality prediction using the DT algorithm and data obtained from a population-based trauma registry, in a Level 1 trauma center. This established model may be useful to determine patients with a high mortality risk, and help improve clinical decision-making in the case of patients with isolated tSAH.

## 2. Materials and Methods

### 2.1. Study Population

Approval was obtained from the institutional review board of the Kaohsiung Chang Gung Memorial Hospital (reference number 201701412B0). This hospital is a Level 1 regional trauma center in southern Taiwan [25,26]. Then, the database of the Trauma Registry System was searched for the diagnostic injury code 852.0 (traumatic subarachnoid hemorrhage) from the International Classification of Diseases, 9th Revision, Clinical Modification (ICD-9-CM). All injury data were coded according to the 1998 version of the Abbreviated Injury Scale (AIS). The AIS is coded by two trauma registrars, who review medical records for written descriptions of injury from radiologists and physicians and do not rely indirectly on ICD-9-CM diagnostic codes. The head AIS scores are presented as the following scores: 1 (minor); 2 (moderate); 3 (serious); 4 (severe); 5 (critical); and 6 (unsurvivable). All adult patients (≥20 years of age) who presented with head trauma and tSAH requiring admission from 1 January 2009 to 31 December 2016, were included in the study. To avoid the confounding effect of injuries of other body regions on the mortality assessment, polytrauma patients [27] with an AIS score ≥3 in any other region of the body were excluded from the study. Thus, the included patients were defined as having isolated tSAH. Of the 1665 identified patients with TBI, 545 patients had isolated tSAH, which included 533 who survived and 12 who died. The following data were retrieved: sex; age; body mass index (BMI); co-morbidities such as diabetes mellitus (DM), hypertension (HTN), coronary artery disease (CAD), congestive heart failure (CHF), cerebral vascular accident (CVA), and end-stage renal disease (ESRD); vital signs, including temperature, systolic blood pressure (SBP), diastolic blood pressure (DBP), heart rate (HR), respiratory rate (RR); shock index (=HR/SBP); Injury Severity Score (ISS); GCS score; AIS score in different regions of the body; white blood cell (WBC) and red blood cell (RBC) count, levels of hemoglobin (Hb), hematocrit (Hct), platelets, blood urine nitrogen (BUN), creatinine (Cr), alanine aminotransferase (ALT), aspartate aminotransferase (AST), sodium (Na), potassium (K), and glucose at the ED; hospital length of stay (LOS); rates of admission to the intensive care unit (ICU); and in-hospital mortality. ISS was expressed as the median and interquartile range (IQR, Q1–Q3).

### 2.2. Decision Tree Classifier

Enrolled patients were divided into a training set and a test set, with a ratio of 7:3. Of the 545 patients with isolated tSAH, 377 and 168 patients, were assigned to the training set and the test set, respectively. The training set was used for predictor discovery and supervised classification to generate a plausible model. The test set was used to test the performance of the model generated in the training sample. The DT classification was performed using classification and regression trees (CART), based on the Gini impurity index using the rpart function in the rpart package in R. The CART analysis searched for the split on the variable that would partition the data into two different groups—a group of mostly ‘1 s’ (people who died) and a group of mostly ‘0 s’ (people who survived) [28,29]. The CART model partitioned the data and assigned a predicted class to each subgroup. With the repetition of the same process on each predictor in the model, the CART identifies the best overall split by iteratively testing all the possible splits and producing the greatest reduction in impurity [30,31]. The CART analysis was performed recursively, in this manner, until the specified stopping criteria were reached, a specified number of nodes were created, or a further reduction in node impurity became impossible [30,31,32]. In order to generate a sequence of simpler trees, each of which is a candidate for the appropriately-fit final tree, the method of “cost-complexity” pruning is used. In this study, the complexity parameter (α), a measure of how much additional accuracy a split must add to the entire tree to warrant additional complexity, was 0.001.

### 2.3. Performance of the Decision Tree Classifier

The accuracy, sensitivity, and specificity of the DT model were calculated. In the test set, stratified 10-fold cross-validation was used to evaluate the predictive power of the models. Briefly, the patients were randomly divided into 10 folds, and the number of patients with an event was approximately equal in all folds. The model was developed using nine folds and validation on the tenth.

### 2.4. Statistical Analysis

We performed the statistical analyses using IBM SPSS Statistics for Windows, version 20.0 (IBM Corp., Armonk, NY, USA) and R 3.3.3 (R Foundation, Vienna, Austria). The primary outcome of the study was in-hospital mortality. Two-sided Fisher’s exact or Pearson’s chi-square tests were used to compare categorical data, with presented odds ratios (ORs) and 95% CIs. The normality of continuous data was examined using the Kolmogorov-Smirnov test. The normally distributed continuous data and non-normally distributed data were analyzed with unpaired Student’s *t*- and Mann-Whitney *U*-tests, respectively, and presented as mean ± standard deviation. *p*-values < 0.05 were defined as statistically significant.

## 3. Results

### 3.1. Characteristics and Outcomes of Patients with Isolated tSAH

No significant differences in sex and comorbidities were observed between the survival and mortality groups (Table 1). The mortality group had significantly higher AIS scores in the head and abdomen regions than the survival group. As shown in Table 2, the mortality group had a significantly higher ISS (median (IQR), 25 (26.3)) than the survival group (15 (2.0)). In addition, the mortality group had a significantly higher HR, glucose, RBC, Na, Cr, and AST level, but lower GCS, DBP, Hb, and Hct levels than the survival group.

### 3.2. Classification by Decision Tree

**Figure 1:**
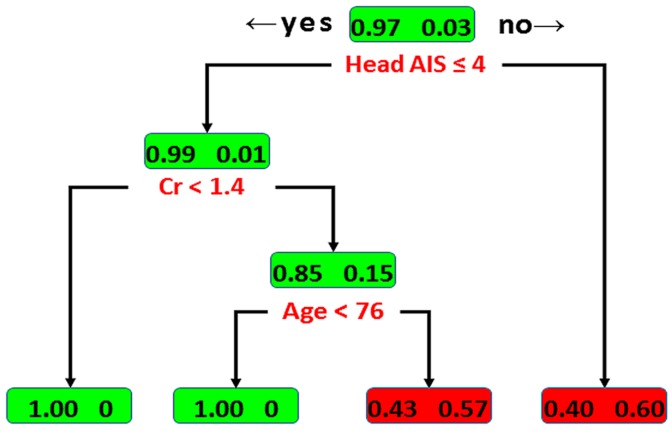
Illustration of the decision tree (DT) model for mortality of patients with isolated traumatic subarachnoid hemorrhage. The boxes denote the percentage of patients with discriminating variables from classification and regression tree (CART) analysis. Those who died and survived are indicated with the fractional number inside the right and left sides of the boxes, respectively.

As shown in Figure 1, in the DT model, the head AIS scorewas identified as the variable of the initial split, with an optimal cut-off value of ≤4. Among patients with a head AIS score >4 (i.e., AIS score = 5 or 6), 60% of the patients with isolated tSAH had fatal outcomes and 40% survived. Among the patients with a head AIS score ≤4, Cr was identified as the variable of the second split, with an optimal cut-off value of <1.4 mg/dL. For this node, all the patients with Cr <1.4 mg/dL survived. The outcome of patients with Cr ≥1.4 mg/dL was determined by an additional predictor—age, with an optimal cut-off value of <76 years; 57% and 43% of patients aged ≥76 had fatal outcomes and survived, respectively. In contrast, all the patients below the age of 76 years of age survived. According to the classification by the DT, two groups of patients with a high risk of fatality were identified. With all the variables in the model, the DT achieved an accuracy of 97.9% (sensitivity of 90.9% and specificity of 98.1%) for the training set. In the test set, the DT achieved an accuracy of 97.7 ± 0.9%, sensitivity of 100.0 ± 0.0%, and specificity of 97.7 ± 0.9%.

## 4. Discussion

In this established DT model, three nodes (head AIS score ≤4, Cr <1.4 mg/dL, and age <76 years) were identified as important determinative variables in the prediction of mortality in patients. In the present study, among the patients with isolated tSAH, 60% of the patients with a head AIS score >4 died, as did 57% of the patients with a head AIS score ≤4, but Cr ≥1.4 and age ≥76 years. However, all the patients who did not fit the above-mentioned criteria survived.

In this DT model, an AIS ≤4 was the first node in predicting fatality in patients. Of the patients with isolated tSAH, in our study, 60% of those with a head AIS = 5 or 6 were likely to die. However, most reports state that tSAH in itself is not a significant prognostic factor of further medical or surgical treatment for mild TBI [11,12,13,14,15]. However, in those head injury patients with an AIS score = 5 (critical) or 6 (unsurvivable), the prognosis is obviously poor. Notably, as per the 1998 version of the AIS, the diagnosis of SAH would be assigned an AIS score = 3. In the upgraded version, AIS score = 4 or 5 would be indicative of the presence or loss of consciousness between 6 and 24 h or >24 h, respectively. Therefore, prolonged time in a state of unconsciousness could also be an important factor in the determination of mortality in patients with isolated tSAH. This is also in agreement with the opinion that the neurological status at admission, after tSAH, reflects early brain injury and is a larger predictor of death [12,13].

Cr <1.4, indicative of renal function, was the second node in the DT model used in the prediction of fatal outcomes. Laboratory values are infrequently included in TBI prognostic evaluation; however, they have been shown to assist in determining patient outcomes [33,34]. For example, patients with SAH are at a high risk of dysnatremia for several weeks following injury [35]. In a multivariate logistic regression model, hypernatremia (defined as sodium >143 mmol/L) was a statistically borderline predictor of mortality [36]. Evidence indicated that posterior hypothalamic lesions trigger renal vasoconstriction by the activation of the renin–angiotensin system, and thereby reduce renal blood flow [37]. A correlation between high plasma renin activity, high urinary catecholamine excretion, and poor patient outcome after SAH has been reported [38]. Furthermore, after aneurysmal SAH, a significant association of renal complications (3.6%, *p* < 0.001) with unfavorable outcomes was reported [11].

The third node in the prediction of mortality patients in this DT model was age <76 years. It is well-known that age, in itself, is an independent predictor of mortality in the case of TBI [39]. The observed increase in mortality begins in the fifth decade of life, with a steep increase occurring at age 70 years in patients with isolated traumatic brain injury [39]. For those with mild-to-moderate TBI, with a GCS score of 9–15, the mortality was twice as high among elderly adults compared to their younger counterparts [39]. Age was also recognized as an independent predictor of mortality in patients with tSAH [9,40], and especially in patients with isolated tSAH [16]. In a previously conducted study, age 58 years was identified as the best threshold for discriminating injury mortality in the case of SAH [41]. In this study, the threshold of age <76 years was selected by the DT algorithm as the best split for further classification.

There are many models, including C4.5 and C5.0, DTs, ID3s, CART, and chi-square automatic interaction detector DTs (CHAIDs), which could be used to construct DT models [26,28]. CART analysis is an innovative DT model in which several predictive variables are crucial in the identification of patients at different levels of risk, in various medical fields, through progressive binary splits, to develop a model for better prediction and clinical decision-making [30,31,32]. Among these methods, CART analysis is conducted based on the combination of nonparametric and nonlinear variables for recursive partitioning analysis [30,31,32]. Approaches with different DT models may provide a model with similar predictive power but with the selection of different kinds of variables as nodes. Determining which tree is the most suitable as a prediction model may depend on the reasonability of the selected node in explaining the predicted outcomes. One advantage of the DT algorithm is its construction does not require any domain knowledge or parameter setting, and is therefore appropriate for exploratory knowledge discovery. The procedure of DT for classifying data based on attributes was different from conventional statistical analysis, which tends to identify the different variables among the compared groups. For example, in this study, three nodes (head AIS score ≤4, Cr <1.4 mg/dL, and age <76 years) were identified as important determinative variables in the prediction of mortality in patients. However, there was even no significant difference of age between the survival and mortality group. Furthermore, mortality group had a significantly higher HR, glucose, RBC, Na, and AST level, in addition to Cr, than the survival group.

One limitation of the study is its relatively small dataset. Therefore, further validation using larger and different datasets may help in the examination of the usefulness of this decision-making model. It has been reported that hemorrhages observed in the basal cistern and Sylvian fissure carry a risk of late deterioration in patients with isolated tSAH [42]. This late deterioration may result from hematoma expansion, which is caused by the abruption of a perforating branch arising from the middle cerebral artery at the time of head injury [42]. Wu et al. also suggested that the presence of tSAH in the basal cisterns or Sylvian fissure on the initial CT scan should be considered as evidence of progressive hemorrhage on the repeat CT scan, and should warrant prompt consideration of neurosurgical consultation [43]. In this study, the lack of important information from CT scans in establishing the DT model may have impaired the predictive power of the constructed model; this is the second limitation of this study.

Furthermore, the prolonged time spent in a state of unconsciousness could have added to the determination of mortality outcomes in the patients in this study. However, this indicates that, before the assignment of the head AIS score, the time of loss of consciousness should be observed for more than 24 h. Therefore, this decision-making algorithm is not capable of providing prediction within 24 h, thus limiting its application. Additionally, as per the 2005 version of the AIS, the diagnosis of SAH would be assigned an AIS score = 2, and the modifier of the loss of conscious code would not be selected if a specific brain injury that can cause unconsciousness is identified [44]. Thus, according to the data used to establish the model in this study, it is preferable to use the state of tSAH with the loss of consciousness >24 h to indicate the first node of the DT model.

According to this DT model, it is not possible to determine the mortality of high-risk patients with very high confidence. However, considering few data are available to help in the identification of high-risk individuals and guide physicians on which patients will likely need further evaluation and treatment, this model may still be helpful in classifying the patients into survival groups (AIS score ≤4 and Cr <1.4 mg/dL as well as AIS score ≤4, Cr ≥1.4 mg/dL, and age <76 years) and groups of high risk to mortality (AIS score >4 and Cr <1.4 mg/dL as well as AIS score ≤4, Cr ≥1.4 mg/dL, and age ≥76 years) with predicted mortality rate. This imperfection also indicated that we have space for improvement in this established DT model. The present study has some other limitations too, the first of which is the selection bias associated with the retrospective study design. Due to the relatively small sample size, the risk factors for mortality may not have been fully assessed. Second, patients who died at the scene or were declared dead upon hospital arrival were not included in this study, leading to further selection bias considering that mortality was the primary outcome. Third, in the absence of a standard protocol for the treatment of patients with isolated tSAH, in this study, we can only assume that the patients included received uniform management by the care-giving physicians, especially considering that there was a lack of important information regarding the administration of anticoagulation and antiepileptic medications. Furthermore, the study was limited to a single center, and the patient injury characteristics and management may vary from those observed at other institutions; this limits the generalizability of the findings.

## 5. Conclusions

The study established a DT model with three nodes (head AIS score ≤4, Cr <1.4, and age <76 years) to predict fatal outcomes in patients with isolated tSAH. The proposed decision-making algorithm may help identify patients with a high risk of mortality.

## Figures and Tables

**Table 1:** Categorical variables of patient sex, co-morbidities, and abbreviation injury scale (AIS) scores in the body regions. CVA: cerebral vascular accident; CAD: coronary artery disease; HTN: hypertension; CHF: congestive heart failure; ESRD: end-stage renal disease DM: diabetes mellitus.

Variables		Total (*n* = 545)	Survival (*n* = 533)	Mortality (*n* = 12)	*p*-Value
Sex	Female	270 (49.5%)	264 (49.5%)	6 (50%)	>0.999
	Male	275 (50.5%)	269 (50.5%)	6 (50%)	
CVA	No	521 (95.6%)	510 (95.7%)	11 (91.7%)	0.421
	Yes	24(4.4%)	23(4.3%)	1 (8.3%)	
CAD	No	528 (96.9%)	516 (96.8%)	12 (100%)	>0.999
	Yes	17 (3.1%)	17 (3.2%)	0 (0%)	
HTN	No	379 (69.5%)	371 (69.6%)	8 (66.7%)	0.762
	Yes	166(30.5%)	162(30.4%)	4 (33.3%)	
CHF	No	537(98.5%)	525(98.5%)	12 (100%)	>0.999
	Yes	8(1.5%)	8(1.5%)	0(0%)	
ESRD	No	538 (98.7%)	526 (98.7%)	12 (100%)	>0.999
	Yes	7(1.3%)	7(1.3%)	0(0%)	
DM	No	469 (86.1%)	458 (85.9%)	11 (91.7%)	>0.999
	Yes	76 (13.9%)	75 (14.1%)	1 (8.3%)	
AIS (Head)	3	452 (82.9%)	449 (84.2%)	3 (25%)	<0.001
	4	79 (14.5%)	77 (14.5%)	2 (16.7%)	
	5	13 (2.4%)	7 (1.3%)	6 (50%)	
	6	1 (0.2%)	0 (0%)	1 (8.3%)	
AIS (Face)	0	410 (75.2%)	401 (75.2%)	9 (75%)	>0.999
	1	48 (8.8%)	47 (8.9%)	1 (8.3%)	
	2	87 (16%)	85 (15.9%)	2 (16.7%)	
AIS (Thorax)	0	502 (92.1%)	491 (92.1%)	11 (91.7%)	0.505
	1	19 (3.5%)	18 (3.4%)	1 (8.3%)	
	2	24 (4.4%)	24 (4.5%)	0 (0%)	
AIS (Abdomen)	0	534 (98%)	523 (98.1%)	11 (91.7%)	0.007
	1	4 (0.7%)	3 (0.6%)	1 (8.3%)	
	2	7 (1.3%)	7 (1.3%)	0 (0%)	
AIS (Extremity)	0	390 (71.6%)	381 (71.5%)	9 (75%)	0.965
	1	52 (9.5%)	51 (9.6%)	1 (8.3%)	
	2	103 (18.9%)	101 (18.9%)	2 (16.7%)	
AIS (External)	0	452 (82.9%)	441 (82.7%)	11 (91.7%)	0.701
	1	93 (17.1%)	92 (17.3%)	1 (8.3%)	

**Table 2:** Continuous variables of patient age, injury characteristic, physiological response, and laboratory data. BMI: body mass index; HR: heart rate; SBP: systolic blood pressure; RR: respiratory rate; GCS: Glasgow Coma Score; ISS: Injury Severity Score; RBC: red blood cell; WBC: white blood cell; Hb: hemoglobin; Hct: hematocrit; BUN: blood urea nitrogen; Cr: creatinine; AST: aspartate aminotransferase; ALT: alanine aminotransferase.

Variables	Total (*n* = 545)	Survival (*n* = 533)	Mortality (*n* = 12)	*p*-Value
Age (years)	58.0 (29.0)	58 (28.5)	72 (50.0)	0.312
BMI	23.9 (3.9)	23.9 (3.9)	22.1 (5.7)	0.149
Shock index	0.6 (0.2)	0.6 (0.2)	0.6 (0.4)	0.183
HR (beats/min)	86 (21.0)	86 (20.0)	107 (23.3)	0.018
SBP (mmHg)	149 (47.0)	148 (46.5)	167 (62.5)	0.247
RR (times/min)	19 (2.0)	19 (2.0)	17.5 (5.5)	0.077
Temperature (°C)	36.4 (0.7)	36.4 (0.7)	36.6 (0.8)	0.740
GCS	15 (2.0)	15 (2.0)	3 (5.5)	<0.001
ISS	13 (5.0)	11 (5.0)	25 (26.3)	<0.001
RBC (10^6^/uL)	4.4 (0.8)	4.4 (0.8)	4.5 (1.2)	0.034
WBC (10^3^/uL)	11.5 (6.8)	11.5 (6.9)	15.4 (10.2)	0.304
Hb (g/dL)	13.2 (2.5)	13.2 (2.5)	13.2 (4.3)	0.032
Hct (%)	39.3 (6.2)	39.3 (6.3)	39 (11.1)	0.035
Platelets (10^3^/uL)	207 (76.0)	207 (77.0)	209 (116.5)	0.247
Glucose (mg/dL)	132 (49.0)	131 (47.0)	195.5 (271.8)	<0.001
Na (mEq/L)	139 (3.0)	139 (3.0)	139 (14.5)	0.016
K (mEq/L)	3.6 (0.6)	3.6 (0.6)	3.8 (1.9)	0.380
BUN (mg/dL)	13 (7.0)	13 (7.0)	18.5 (18.8)	0.081
Cr (mg/dL)	0.8 (0.4)	0.8 (0.4)	1.1 (0.9)	0.005
AST (U/L)	32 (19.0)	32 (18.0)	49 (74.0)	0.003
ALT (U/L)	25 (18.0)	25 (18.5)	25.5 (29.8)	0.202
